# Urinary comprehensive genomic profiling predicts urothelial carcinoma recurrence and identifies responders to intravesical therapy

**DOI:** 10.1002/1878-0261.13530

**Published:** 2023-10-05

**Authors:** Goran Rac, Hiten D. Patel, Christopher James, Shalin Desai, Vincent M. Caruso, Daniel S. Fischer, Peter S. Lentz, Ceressa T. Ward, Brian C. Mazzarella, Kevin G. Phillips, Chirag Doshi, Vincent T. Bicocca, Trevor G. Levin, Alan J. Wolfe, Gopal N. Gupta

**Affiliations:** ^1^ Department of Urology Loyola University Medical Center Maywood IL USA; ^2^ Department of Urology, Fienberg School of Medicine Northwestern University Evanston IL USA; ^3^ Convergent Genomics, Inc. South San Francisco CA USA; ^4^ Department of Microbiology and Immunology Loyola University Chicago Maywood IL USA; ^5^ Department of Radiology Loyola University Medical Center Maywood IL USA; ^6^ Department of Surgery Loyola University Medical Center Maywood IL USA

**Keywords:** Bacillus Calmette‐Guérin, bladder cancer, genomics, intravesical instillation, personalized medicine, risk assessment

## Abstract

Intravesical therapy (IVT) is the standard of care to decrease risk of recurrence and progression for high‐grade nonmuscle‐invasive bladder cancer. However, post‐IVT recurrence remains common and the ability to risk‐stratify patients before or after IVT is limited. In this prospectively designed and accrued cohort study, we examine the utility of urinary comprehensive genomic profiling (uCGP) for predicting recurrence risk following transurethral resection of bladder tumor (TURBT) and evaluating longitudinal IVT response. Urine was collected before and after IVT instillation and uCGP testing was done using the UroAmp™ platform. Baseline uCGP following TURBT identified patients with high (61%) and low (39%) recurrence risk. At 24 months, recurrence‐free survival (RFS) was 100% for low‐risk and 45% for high‐risk patients with a hazard ratio (HR) of 9.3. Longitudinal uCGP classified patients as minimal residual disease (MRD) Negative, IVT Responder, or IVT Refractory with 24‐month RFS of 100%, 50%, and 32%, respectively. Compared with MRD Negative patients, IVT Refractory patients had a HR of 10.5. Collectively, uCGP enables noninvasive risk assessment of patients following TURBT and induction IVT. uCGP could inform surveillance cystoscopy schedules and identify high‐risk patients in need of additional therapy.

## Graphical abstract

This study used genomic profiling to detect mutations associated with urothelial carcinoma in patient urine samples collected before and after intravesical therapy. Genomic profiles informed an algorithmic assessment of minimal residual disease, which accurately predicted recurrence risk after surgery and quantified molecular response to therapy. These findings demonstrate the utility of genomics to inform personalized risk stratification.

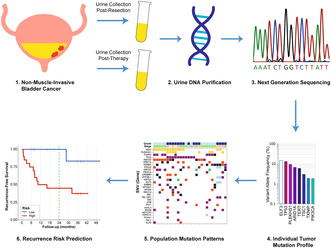

**Abbreviations**
BCG: Bacillus Calmette‐Guérin
CIS: carcinoma *in situ*
GDB: genomic disease burden
HR: hazard ratio
IVT: intravesical therapy
MRD: minimal residual disease
NMIBC: nonmuscle‐invasive bladder cancer
RFS: recurrence‐free survival
TURBT: transurethral resection of bladder tumor
UC: urothelial carcinoma
uCGP: urinary comprehensive genomic profiling
VAF: variant allele frequency

## 1 Introduction

The standard of care for patients with nonmuscle‐invasive bladder cancer (NMIBC) involves transurethral resection of bladder tumor (TURBT) followed by induction intravesical therapy (IVT) for those at intermediate or high risk of recurrence [1]. Despite this well‐established treatment paradigm, recurrence and progression rates remain high, with 50–70% of patients recurring [2, 3] and as many as 45% progressing to muscle‐invasive bladder cancer (MIBC), radical cystectomy, or metastatic disease within 5 years [4].

Surgical and therapeutic options have incrementally improved over time, but risk stratification remains a challenge. Repeat TURBT may benefit some patients [3, 5] and improved utilization of post‐TURBT intravesical chemotherapy or Bacillus Calmette‐Guérin (BCG) maintenance have modestly improved outcomes [6, 7, 8]. For NMIBC recurrence after IVT, additional IVT regimens, pembrolizumab and nadofaragene firadenovec [9, 10, 11], and ongoing clinical trials are options [12, 13]. For some, radical cystectomy may be the best remaining option, and patients who receive timely cystectomy within 24 months of diagnosis experience a significant survival advantage [14, 15]. As treatment options expand, so does the need for better risk stratification to guide treatment utilization.

High recurrence rates suggest the presence of minimal residual disease (MRD) following treatment. Prognostic MRD detection could help with patient risk stratification, as is common in hematologic malignancies and other solid tumors, but it has yet to be routinely incorporated into urothelial carcinoma (UC) treatment paradigms [16]. Instead, the gold standard for post‐TURBT surveillance is a combination of cystoscopy and urine cytology to detect an already recurred tumor—and is fraught with poor sensitivity [17, 18]. Clinical risk factors are limited in their ability to predict which patients will have a durable response after IVT, requiring less intensive surveillance, and which patients will be refractory to IVT and could benefit from immediate repeat TURBT, accelerated second‐line therapy, or more timely counseling on radical cystectomy. These patients may then miss their window of opportunity for curative treatment before progression while waiting for their cancer to visually recur.

One previously studied test to risk‐stratify response to BCG therapy is fluorescence *in situ* hybridization (FISH) [19, 20, 21]. However, FISH suffers from many of the same limitations of cytology, namely poor overall sensitivity [22], dependence on intact cells, and delivery of a binary (nonquantitative) readout. At the same time, FISH lacks the key benefit provided by cytology: pathologic grading. In contrast, urinary comprehensive genomic profiling (uCGP), which quantifies diverse types of somatic tumor mutations from urine‐derived DNA, detects MRD with high sensitivity and uses genomic features associated with stage and grade to support risk stratification [23]. Many genes and mutation types measured within the genomic profile are also associated with drug response or are direct targets of next‐generation drugs. Therefore, we aimed to evaluate baseline and longitudinal uCGP in a prospective cohort of NMIBC patients to assess TURBT efficacy, IVT response, and predict 24‐month recurrence after IVT.

## 2 Materials and methods

### 2.1 Study population and design

Institutional Review Board (WCG, IRB00000533; LUCHSC, IRB00008315) approval was obtained prior to study initiation. The study methodologies conformed to the standards set by the Declaration of Helsinki. Adult patients (≥ 18 years of age) diagnosed with pathologically confirmed NMIBC with UC histology at Loyola University Medical Center (Maywood, IL) or Golden Gate Urology (Berkeley, Oakland, and San Francisco, CA) from January 2019 to April 2021 gave written informed consent and were prospectively enrolled. The cohort includes AUA/SUO intermediate‐ and high‐risk patients [1], all of whom were BCG‐naïve at the time of entry into the study and who elected to undergo primary BCG induction. Patients with a prior history of pelvic radiation, history of IVT, or a non‐UC histologic subtype were excluded. Baseline demographics (e.g., age, sex, and race), smoking status, history of prior UC, and family history of UC were obtained. If a patient had a history of prior UC, tumor grade and location were recorded. Clinical and pathologic data also included stage of the primary tumor, presence of carcinoma *in situ* (CIS), and type of IVT. This work was performed in accordance with STARD/REMARK best practices for diagnostic accuracy studies [24, 25].

### 2.2 Intravesical therapy

All patients underwent a 6‐week induction course of IVT using either BCG or gemcitabine. Intravesical BCG was administered weekly according to the protocol outlined by SWOG [7]. Maintenance IVT was administered at the discretion of the treating physicians and dependent on the availability of BCG. Maintenance IVT regimens generally consisted of three weekly treatments at three and 6 months and then every 6 months for a total of 36 months. BCG dose reductions were allowed at the discretion of the treating physician.

### 2.3 Urinary comprehensive genomic profiling

Urine samples were collected immediately prior to initiation and after completion of IVT. All collected urine samples were preserved in Enhanced Preservation Media (Convergent Genomics, South San Francisco, CA, USA) and provided to the sponsor (Convergent Genomics) in a blinded fashion, ensuring the clinical status of samples was unknown by the sponsor laboratory or data analysis personnel during sample processing.

Urinary comprehensive genomic profiling was performed with the Clinical Laboratory Improvement Amendments (CLIA)‐validated UroAmp™ platform (Convergent Genomics). UroAmp uses next‐generation sequencing to detect six classes of mutations. Five of these—single‐nucleotide variants (SNVs), small insertion‐deletions (INDELs), targeted gene‐level copy‐number variants (CNVs), microsatellite instability (MSI), and copy‐neutral loss of heterozygosity (LOH)—are assayed across a 60‐gene panel, while the sixth mutation type is whole‐genome aneuploidy. UroAmp is a tumor‐naïve test. Detected mutations are annotated using publicly available data sources, including dbSNP, 1000 Genomes Project, The Cancer Genome Atlas, Sanger/COSMIC, and AARC Project GENIE [26, 27, 28, 29, 30]. Mutation level impact is further assessed by Mutation Assessor and SNPeff [31, 32]. Mutation profiles serve as input features to machine‐learned algorithms disease and molecular grade prediction. UroAmp risk algorithms are calculated independent of any clinical features. UroAmp testing methodology was performed as previously described [33].

### 2.4 Patient monitoring

Patients were monitored during IVT treatment according to standard‐of‐care practice outlined in National Comprehensive Cancer Network guidelines [34]. Consistent with American Urological Association (AUA) guidelines [1], surveillance of NMIBC high‐risk patients was performed by cystoscopy and cytology at 3‐month intervals for 2 years and 3‐ to 6‐month intervals thereafter. Upper tract and abdominal/pelvic imaging baselines were established and repeated every 1–2 years or as clinically indicated. Observed recurrences were confirmed pathologically.

### 2.5 Outcomes and risk algorithms

The primary endpoint of the study was 24‐month recurrence‐free survival (RFS). Secondary analyses included quantification of genomic features such as mean variant allele frequency (VAF) and genomic disease burden (GDB). Genomic disease burden is a percentile ranking of the sum of VAF for a urine specimen. As described previously, the percentile rank underlying GDB was determined from a training cohort to generate a representative empirical distribution [23]. Each test sample was then ranked against that distribution to obtain its GDB percentile.

The independent variables of interest were the UroAmp recurrence risk algorithms for (a) baseline pre‐IVT recurrence risk and (b) longitudinal response comparing post‐IVT uCGP to pre‐IVT uGCP. Training and validation of the UroAmp recurrence risk algorithm were previously described [23]. Briefly, recurrence risk classification of ‘high’ or ‘low’ is determined according to an algorithm trained to evaluate the genes mutated and their VAF. Here, UroAmp recurrence risk prediction was applied to baseline urine collections taken following TURBT but before IVT induction.

Longitudinal urine collections (before and after IVT) were used to determine IVT response groups. Patients were stratified into distinct groups depending upon the genomic signatures identified over the course of treatment by comparing the post‐IVT uCGP to the pre‐IVT uCGP. Predicted response groups are defined as follows:

1. *MRD Negative*—Patients are UroAmp low recurrence risk at both the baseline urine collection and the post‐IVT collection.
2. *IVT Responder*—Patients are UroAmp high recurrence risk at their baseline urine collection and UroAmp low recurrence risk following IVT or demonstrated a decrease of at least 0.25 (on a scale of 0–1) in their algorithm score between pre‐ and post‐IVT collections.
3. *IVT Refractory*—Patients are UroAmp high recurrence risk in the post‐IVT collection or demonstrated an increase of at least 0.25 (on a scale of 0–1) in their recurrence risk algorithm score between pre‐ and post‐IVT collections.

### 2.6 Statistical analysis

The association between baseline pre‐IVT recurrence risk and longitudinal IVT response was assessed by Kaplan–Meier RFS analysis, which was performed with the *survival* package in R with primary endpoint at 24 months [35]. Cox proportional hazards regression models estimated hazard ratios (HR) and were done in Python with the *lifelines* package [36]. All other statistical analyses were done in Python using the *SciPy* and *statsmodels* packages [37, 38].

## 3 Results

### 3.1 Cohort characteristics

A total of 36 patients met pre‐defined inclusion criteria. Age, race, sex, smoking status, prior UC history, and family history are detailed in Table 1. High‐grade (HG) disease was present in 35 (97.2%) patients, and primary tumor stage included Ta (*n* = 14), T1 (*n* = 19), and CIS only (*n* = 3). IVT included intravesical BCG for 33 patients and gemcitabine for three. Sample usage is detailed in Fig. S1, with analyzable test results for 33 pre‐IVT samples, 32 post‐IVT samples, and 29 matched pre‐ and post‐IVT samples. The median length of follow‐up was 19 months.

**Table 1:** Patient demographics, clinical, and pathologic factors. IQR, interquartile range.

	Recurrence (*n* = 13)	Nonrecurrence (*n* = 23)
Age, median (IQR)	72 (6)	69 (17)
Sex, *n* (%)		
Male	13 (100%)	18 (78.3%)
Female	0 (0%)	5 (21.7%)
Race, *n* (%)		
Asian	2 (15.4%)	1 (4.4%)
Black	0 (0%)	4 (17.4%)
Caucasian	11 (84.6%)	16 (69.6%)
Hispanic	0 (0%)	1 (4.4%)
Other	0 (0%)	1 (4.4%)
Smoking status, *n* (%)		
Current	1 (7.7%)	2 (8.7%)
Past	8 (61.5%)	12 (52.2%)
Never	4 (30.8%)	9 (39.1%)
Family history of UC, *n* (%)		
Yes	0 (0%)	0 (0%)
No	13 (100%)	23 (100%)
Primary tumor grade^a^, *n* (%)		
Low grade	1 (7.7%)	0 (0%)
High grade	12 (92.3%)	23 (100%)
Primary tumor stage^a^, *n* (%)		
Ta	3 (23.1%)	11 (47.8%)
T1	7 (53.8%)	12 (52.2%)
CIS alone	3 (23.1%)	0 (0%)
CIS presence^a^, *n* (%)		
Yes	5 (38.5%)	8 (34.8%)
No	8 (61.5%)	15 (65.2%)
History of UC^b^, *n* (%)		
Low‐grade bladder	2 (15.4%)	0 (0%)
High‐grade bladder	0 (0%)	1 (4.4%)
Upper tract	1 (7.7%)	0 (0%)
Induction IVT regimen, *n* (%)		
BCG	12 (85.7%)	21 (89.5%)
Gemcitabine	1 (14.3%)	2 (10.5%)

^a^ Pathology for tumor prior to IVT.

^b^ History of UC prior to study enrollment.

### 3.2 Baseline uCGP assesses TURBT efficacy and predicts recurrence‐free survival

**Fig. 1:**
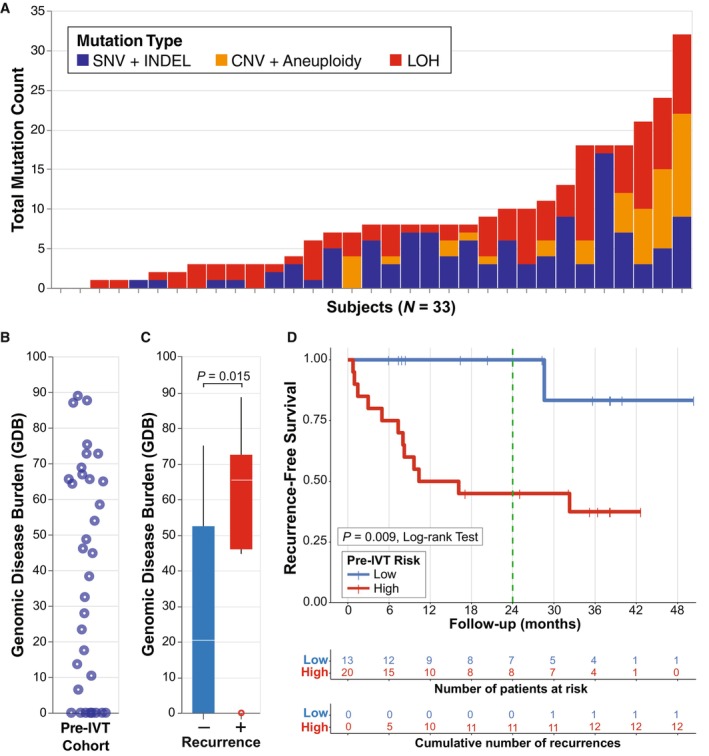
Urinary comprehensive genomic profiling detects MRD to predict recurrence risk following TURBT. Prior to beginning IVT, uCGP was performed on NMIBC patients following curative‐intent TURBT. (A) Cumulative mutation counts for all pre‐IVT subjects (*N* = 33) are shown. Single nucleotide variant (SNV) and insertion/deletion (INDEL) mutations are grouped together. Copy‐number variation (CNV) and aneuploidy events are grouped together. (B) A strip plot demonstrates the variability of GDB following TURBT. (C) Box plot of mean GDB in patients that did and did not recur. Statistical significance was determined by the Mann–Whitney *U*‐test. (D) RFS by UroAmp pre‐IVT low and high recurrence risk groups. The vertical green line indicates the primary study endpoint of 24 months.

Baseline pre‐IVT mutation profiles for 33 patients with eligible samples demonstrated significant diversity across the cohort in terms of mutation type, number, and GDB (Fig. 1A,B). Blinded predictions of baseline recurrence risk using the previously described UroAmp recurrence risk algorithm [23] stratified the cohort into low (*n* = 13) and high (*n* = 20) recurrence risk groups. A total of 13 (39%) patients experienced recurrence. Patients who recurred demonstrated significantly higher GDB compared with those who did not (Fig. 1C). Patients classified by uCGP as high‐risk had a 24‐month Kaplan–Meier RFS of 45% compared with 100% for patients classified as low‐risk (*P* = 0.009, log‐rank test, Fig. 1D). Over the entire course of surveillance follow‐up, only one recurrence (8%) was observed (28 months) among the low‐risk patients, and 12 total recurrences (60%) were observed among the high‐risk patients (Fig. 1D). Among high‐risk patients, GDB further correlates with risk of recurrence, as high GDB patients (defined as the top‐quartile of GDB) demonstrated an RFS of 0% (Fig. S2). Of the eight high‐risk patients who did not have a cystoscopically confirmed recurrence, four (50%) had concomitant CIS at initial diagnosis.

### 3.3 Longitudinal uCGP identifies IVT responders

Similar to baseline uCGP, we found that post‐IVT mutation profiles showed significant diversity, with some patients exhibiting clear changes to their mutation profiles over the course of treatment. Representative examples of SNV profiles and GDB scores from patients classified as MRD Negative, IVT Responder, and IVT Refractory are presented in Table 2. The MRD Negative example has a low GDB score (10.7) driven by a single low‐VAF SNV detected prior to induction and no mutations detected following IVT. The IVT Responder example has a modest GDB (53.2) due to multiple mutations with moderate VAFs prior to IVT. Following IVT, only two of these mutations remain and with reduced VAFs. Finally, the IVT Refractory example has a high GDB before (75.2) and after (85.2) IVT, with already high‐VAF mutations exhibiting increased intensity following IVT.

**Table 2:** Longitudinal MRD assessment identifies responders to IVT. Following curative‐intent TURBT, uCGP was performed on patients pre‐ and post‐IVT. Based on these longitudinal MRD assessments, the following response groups were established: MRD Negative, IVT Responder, and IVT Refractory. The characteristics of these response groups are detailed with UroAmp mutation profiles and GDB. Tumor mutations are color‐coded to facilitate identification across collections. Solid bars indicate single‐nucleotide variants (SNVs) and open bars indicate insertion‐deletions (INDELs). Recurrence status at the last follow‐up is included.

Response group	MRD Negative	IVT responder	IVT refractory
Group description	Low urinary GDB following TURBT	High urinary GDB following TURBT and responds to IVT	High urinary GDB following TURBT and does not respond to IVT
UroAmp mutation profile & GDB	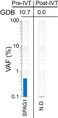	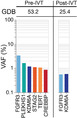	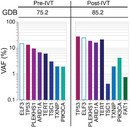
Recurrence status	No recurrence (40 months)	No recurrence (38 months)	HG T1 (8 months)

**Fig. 2:**
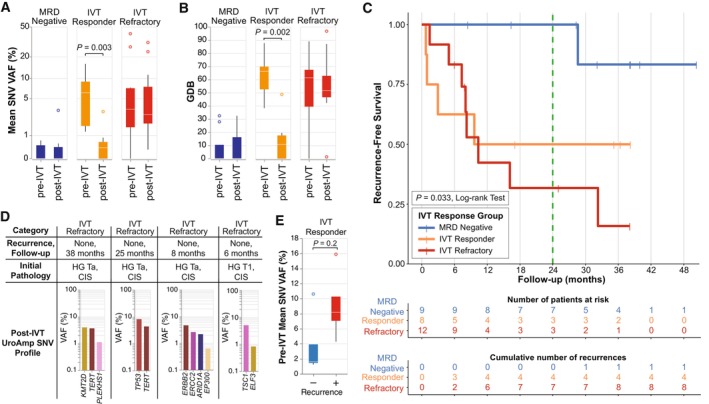
Longitudinal MRD detection stratifies patients by IVT response and predicts recurrence risk. (A) Box plots demonstrate mean SNV VAF and (B) mean GBD quantified for each response group (MRD Negative, Responder, and Refractory) prior to and following IVT. Statistical significance was determined by the Mann–Whitney *U*‐test. (C) RFS by IVT response group. The vertical green line indicates the primary study endpoint of 24 months. (D) Patients in the ‘IVT Refractory’ response group who do not have diagnosed recurrences are shown with follow‐up time, initial pathology, and post‐IVT uCGP tumor mutation profile. (E) Box plot of pre‐IVT mean SNV VAF for patients in the responder group are shown by recurrence status.

Of the 29 patients with pre‐ and post‐IVT uCGP assessments, nine (31%) were classified as MRD Negative, eight (28%) were classified as IVT Responders, and 12 (41%) were classified as IVT Refractory. Multiple measures of residual disease, such as GDB and mean SNV VAF, distinguished the three groups with only the IVT Responders demonstrating significant changes in tumor burden over the course of IVT (Fig. 2A,B). Kaplan–Meier 24‐month RFS was 100%, 50%, and 32% for the MRD Negative, IVT Responder, and IVT Refractory groups, respectively (*P* = 0.033, log‐rank test) (Fig. 2C). At 36 months, the RFS of IVT Refractory patients fell to 16% and, over the entire course of follow‐up, one (11%) MRD Negative patients experience recurrence (28 months). All IVT Responder group recurrences occurred within 12 months with no additional events thereafter (32‐month mean follow‐up).

Chart review of the four IVT Refractory patients who did not have a diagnosed recurrence revealed that all of them had CIS present at initial diagnosis. Post‐IVT uCGP of these four patients revealed modest disease burdens composed of high‐risk mutation signatures, including *TERT*, *TP53*, and *ERBB2* (Fig. 2D), consistent with the possibility of visually occult residual CIS. We further investigated the IVT Responder group for clinical and genomic features correlating with recurrence status. Only two patients in the IVT Responder group had the initial presence of CIS at diagnosis, and both recurred. uCGP analysis revealed that the average preinduction mutation intensity (mean SNV VAF) of recurrence‐positive patients was ~5 times that of recurrence‐negative patients within the IVT Responder group (Fig. 2E). Finally, we assessed ARID1A mutations which were previously shown to be more prevalent in patients with BCG recurrence [39, 40]. We observed ARID1A mutation enrichment of 1.7‐fold in IVT Refractory versus IVT Responder patients. Among IVT Refractory tumors, 80% demonstrated ARID1A clonal expansion through induction, with an average VAF increasing 3.7‐fold.

### 3.4 uCGP identifies targeted therapy eligibility for IVT refractory patients

We examined the ability of uCGP to detect mutations that would identify patients as candidates for second‐line, targeted therapy clinical trials. Repeated mutations in *ERBB2* (*HER2*) and *ERBB3*, which can act as oncogenes both alone and as an oncogenic heterodimer [41], were found in 25% and 17% of IVT Refractory patients, respectively (Table 3). *PIK3CA* and *TSC1* were also repeatedly mutated and enriched specifically in IVT Refractory patients, each presenting in 25% of patients in the group. Though *FGFR3* is one of the most common mutations in UC and a clear candidate gene for targeted therapies, it is preferentially enriched in LG disease [28, 33]. Consistent with this, *FGFR3* mutations were relatively rare in our cohort (6%), and they were never observed in IVT Refractory patients. Other targeted therapy candidates, including *ERCC2*, were identified in the overall cohort but not in the IVT Refractory group.

**Table 3:** Targeted therapy trial eligibility identified by uCGP.

Druggable target	Prevalence in cohort (*n* = 36)	Prevalence in IVT refractory patients (*n* = 12)	Drug name	Clinical Trail ID
ERBB2 (HER2)	6 (17%)	3 (25%)	Disitamab vedotin^a^ Trastuzumab^a^	NCT05723991 NCT05318339
PIK3CA	4 (11%)	3 (25%)	Alpelisib Copanlisib	NCT04589650 NCT05687721
TSC1	3 (8%)	3 (25%)	*nab*‐Sirolimus^a^	NCT05103358
ERBB3	2 (6%)	2 (17%)	Neratinib	NCT03065387, NCT01953926
FGFR3	2 (6%)	0 (0%)	Erdafitinib^a^	NCT05564416
ERCC2	1 (3%)	0 (0%)	Cetrelimab Avelumab^a^	EUCTR2020‐004506‐64‐ES NCT05568407, NCT05327530

^a^ Denotes drugs with many ongoing trials in bladder cancer and other solid tumors.

### 3.5 Cox proportional hazards regression with uCGP and clinical risk factors

**Fig. 3:**
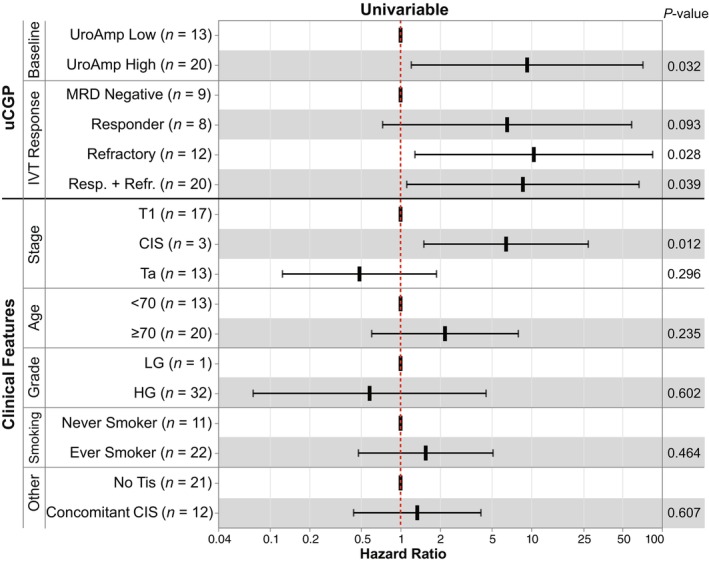
Urinary comprehensive genomic profiling predicted recurrence risk. Baseline (*n* = 33) and longitudinal (*n* = 29) NMIBC cohorts were analyzed for recurrence risk. Univariable Cox proportional‐hazard regression analyses of UroAmp recurrence risk and IVT response groups as well as clinical risk factors are shown with confidence intervals. *P*‐values were determined by the Wald test.

Univariable Cox proportional hazards regression of baseline pre‐IVT uCGP recurrence risk showed high‐risk patients had a HR 9.3 (95% CI 1.2–71.5, *P* = 0.032) relative to low‐risk patients (Fig. 3, Table S1). For longitudinal IVT response groups, IVT Responder had a HR 6.5 (95% CI 0.7–58.6, *P* = 0.093) and IVT Refractory had a HR 10.5 (95% CI 1.3–84.9, *P* = 0.028) relative to MRD Negative. The difference between IVT Responder and IVT Refractory groups was limited by sample size and not statistically significant (*P* = 0.45). Clinical risk factors of age, smoking history, history of UC, concomitant CIS, and stage (T1 vs Ta) were not significantly associated with recurrence (Fig. 3, Table S1). However, presence of CIS alone (*n* = 3) was associated with recurrence with HR 6.4 (95% CI 1.5–27.3, *P* = 0.012). AUA/SUO intermediate‐ and high‐risk patients had recurrence rates of 50% and 39%, respectively (Table S2).

## 4 Discussion

Currently, patients with intermediate‐ or high‐risk NMIBC and their physicians face treatment dilemmas at three critical points of care following curative‐intent TURBT. First, the extent of surgical success is highly variable and limited options exist to assess efficacy. Second, failure of residual tumor to respond to IVT is common and options for assessing response are limited [42]. Third, post‐treatment surveillance modalities (cystoscopy) are burdensome and not prognostic, with risk assessment based primarily on the pathology of cauterized tissue fragments. Risk stratification by initial tissue pathology has limitations, and prognostic value may diminish as time from original TURBT increases due to exposure to the selective pressures of resection and IVT. AUA NMIBC risk groups based on clinical factors including primary stage and grade, tumor size, and recurrence history often guide the decision to administer induction IVT [1]. However, despite this stratification and receipt of IVT by intermediate‐ and high‐risk patients, recurrence rates remain high. For patients with tumors that do not respond to IVT and progress to MIBC, survival rates are 35–40% [43, 44]. Predictive information about an individual's tumor status following curative‐intent TURBT can help guide treatment decisions. In the present study, we demonstrate the potential of uCGP to support clinical decision‐making at each of these critical treatment time points.

Previous work has shown the utility of uCGP for the initial diagnosis of UC in patients with hematuria and for the prediction of long‐term recurrence risk in UC surveillance patients [23]. Here, we present the first longitudinal assessment of IVT response based on uCGP. Baseline MRD detection following TURBT revealed a remarkable heterogeneity of mutation signal among the study cohort. This is seen after visually complete TURBT performed by experienced surgeons. These data provide striking context for tumor variability, with some tumors being much harder to achieve full molecular resection. We demonstrate the power of this baseline mutation signal to serve as the basis for molecular risk stratification. Patients who experience recurrence have higher GDBs after TURBT than those who do not, and when baseline UroAmp recurrence‐risk prediction algorithms are applied, high and low recurrence risk patient populations can be clearly distinguished. RFS and hazard analyses show high‐risk patients are > 9 times more likely to recur than low‐risk, indicating that the extent to which TURBT removes mutations is the most significant independent risk factor in a patient's recurrence risk. For those identified as high‐risk, a host of alternative management options can be considered, including repeat TURBT, enhanced cystoscopy (blue light or narrow band imaging), or upper tract evaluation.

Two approaches can be used to detect MRD during surveillance: tumor‐informed and tumor‐naive. A tumor‐informed approach uses the mutations detected in the resected tumor to inform what mutations to prioritize during surveillance. A tumor‐naïve approach uses learned mutation patterns that correlate with disease and then looks for those patterns during surveillance. Both approaches can be effective [45, 46] and have distinct advantages depending upon cancer type, staging, and patient clinical characteristics. We believe the largely unique biology of UC generally supports a tumor‐naïve approach. Previous work has shown that UC‐grade progression can occur with low mutation homology, either from true tumor progression or de‐novo growth from a related premalignant field [47]. Furthermore, exome sequencing studies of multifocal metachronous and synchronous UC tumors showed mutation homology as low as 6% and as high as 53% between studied tumors [48]. Therefore, recurrence and progression risk are not derived strictly from the visually apparent tumor removed during TURBT. Notably, the uCGP technology used here was previously validated to accurately genotype bladder tumors from urine collections taken prior to TURBT [33], so the same technology could be used in a tumor‐informed MRD approach if a urine sample is collected prior to resection. However, that same study demonstrated the mutation heterogeneity derived from bladder field defects that appear concurrent with a primary tumor. Here, we further demonstrate the power of a tumor‐naïve approach where mutations derived from residual tumor, independent malignancy, or premalignant field are all considered.

Longitudinal assessment of MRD throughout treatment has the potential to provide further risk stratification of NMIBC patients [20]. Using uCGP, we were able to identify three response groups depending upon their molecular disease burdens before and after IVT. Patients identified as IVT Refractory did not see their MRD signal respond to IVT and recurred at the highest rate, but only two of these patients (17%) recurred within the traditional 6‐month cutoff to be considered clinically BCG‐refractory [49]. Four more patients (33%) recurred in the subsequent 6 months, and these patients would normally be considered BCG‐relapsing, despite their tumors showing no evidence of response to IVT induction based on uCGP. On the whole, the UroAmp‐defined IVT Refractory cohort demonstrated a RFS of just 16% over the entire course of surveillance. Notably, the patients who did not have diagnosed recurrences all presented with concomitant CIS during their initial resection. The residual disease signal in these patients begs the question whether their residual disease is simply more difficult to visually diagnose by cystoscopy. Expanded studies with enhanced imaging surveillance in this group will be required to better address these questions. It is also acknowledged that BCG maintenance was offered to all study subjects at three and 6 months and then every 6 months for a total of 36 months, but urine was not collected before or after maintenance regimens. This study's refractory genomic risk assessment is based upon response through primary induction and cannot rule out the potential for delayed maintenance therapy benefit among those without visually confirmed recurrence.

Patients identified as IVT Responders still recurred at high rates. Preliminary evidence suggests that tumor burden at the start of IVT may be the distinguishing feature that separates IVT Responder patients that recur from those who do not. Many of these high tumor‐burden IVT Responders had their recurrences identified within the first 6 months of surveillance. Traditionally, this would classify these patients as clinically BCG‐refractory [49]. We show that despite the residual tumor in the bladder, these patients had a favorable reduction in mutation abundance in response to IVT. In these patients, extended induction dosing beyond the standard 6 weeks may provide benefit and should be studied.

**Fig. 4:**
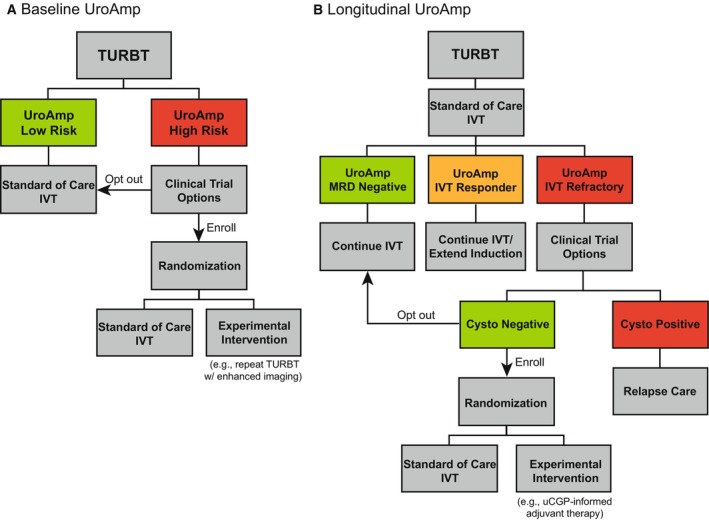
Proposed urinary comprehensive genomic profiling‐informed clinical trial design. (A). Baseline UroAmp can inform early intervention trial design following curative‐intent TURBT of NMIBC. Here, UroAmp identifies high‐recurrence risk patients who are most likely to benefit from further surgical intervention. An appropriate trial may randomize these patients to receive standard‐of‐care therapy (control) or an experimental intervention such as repeat TURBT using an enhanced imaging technology followed by standard‐of‐care therapy. (B). Longitudinal UroAmp can inform ethical trial design following standard‐of‐care IVT induction. Here, IVT Responders can be identified for trials aiming to potentiate the efficacy of induction or maintenance regimens (e.g., extended induction). IVT Refractory patients can be identified for trials of second‐line therapies. Gene targets identified by uCGP can inform appropriate targeted therapy trials. Critically, the proposed trial designs offer ethical control arms and do not delay opportunities for radical cystectomy for patients who fail either control or experimental arms.

Longitudinal MRD detection can be used to better inform clinical trial design. Previous work studied longitudinal FISH testing to identify ‘molecular BCG failure’ [19]. There, investigators proposed that patients identified as molecular failures at their final BCG instillation could be offered enrollment in a clinical trial. Our results suggest uCGP‐mediated MRD detection may be used in a similar manner. For example, patients identified as UroAmp high‐risk at their baseline uCGP could be eligible for studies evaluating the effect of immediate repeat TURBT (perhaps guided by enhanced imaging) on induction durability and long‐term recurrence (Fig. 4A). Postinduction uCGP would then identify high‐risk patients who respond to therapy. IVT Responders on longitudinal assessment could be considered for extended induction. Patients identified as IVT Refractory can be counseled that they have a ~ 50% chance of recurring within 12 months if they proceed with standard‐of‐care IVT. These patients may be offered the opportunity to enroll in therapeutic clinical trials (Fig. 4B). Alternatively, some may instead benefit from immediate radical cystectomy. Because uCGP identifies individual gene mutations, patients with mutations in therapeutically targetable genes may be preferentially selected for therapies (Table 3).

The most significant limitation of this study was cohort size. This study was powered for general assessment of MRD detection and its longitudinal relationship with recurrence. However, it was not powered to evaluate progression, which is a critical consideration but would require larger sample size and follow‐up. The limited cohort size also restricted our ability to assess individual molecular relationships. For example, previous studies have identified ARID1A mutations as indicators of BCG failure [39, 40]. We see a similar pattern emerge, but the cohort size constrains statistical confidence. Furthermore, the cohort consisted largely of Caucasian males, and thus, further studies are needed to assess the generalizability of these findings in other patient populations. Urine samples were not collected during postinduction maintenance cycles and so this study is unable to assess the potential impact of molecular response during maintenance. Finally, most of the patients underwent induction with BCG, but some patients received gemcitabine induction due to BCG shortages.

## 5 Conclusion

Urinary comprehensive genomic profiling enables noninvasive risk assessment of patients following TURBT and induction IVT based on MRD. The baseline uCGP high‐risk group experienced a ninefold higher risk of recurrence compared with the low‐risk group. Furthermore, our findings support the use of longitudinal uCGP to assess TURBT efficacy to inform surveillance intensity, evaluate IVT response, and identify IVT refractory patients prior to recurrence or progression to determine targeted therapy candidacy for second‐line therapy or clinical trials. Ongoing studies are expected to support the generalizability of these findings and expand our understanding of the molecular underpinnings of IVT response.

## 7 Conflict of interest

VMC, DSF, PSL, CTW, BCM, KGP, VTB, and TGL report being employees and shareholders of Convergent Genomics. AJW discloses membership on the advisory boards of Urobiome Therapeutics and Pathnostics and funding from Pathnostics, VB Tech, the Craig H. Neilsen Foundation, and NIH. All other authors have no disclosures.

## 8 Author contributions

GR, TGL, AJW, and GNG contributed to the conceptualization. GR, HDP, CJ, SD, CD, VMC, DSF, KGP, CTW, and BCM contributed to the data curation. GR, HDP, KGP, VMC, DSF, VTB, and TGL contributed to the formal analysis. TGL, VTB, and KGP contributed to the funding acquisition. GR, HDP, TGL, CJ, PSL, VTB, and BCM contributed to the investigation. GR, HDP, CJ, BCM, KGP, TGL, and GNG contributed to the methodology. KGP, VMC, and DSF contributed to the software. AHW and GNG contributed to the supervision. VTB, VMC, and KGP contributed to the visualization. GR, VTB, KGP, and CTW contributed to the writing—original draft. GNG, HDP, VMC, BCM, TGL, and AJW contributed to the writing—review and editing.

9

### 9.1 Peer review

The peer review history for this article is available at https://www.webofscience.com/api/gateway/wos/peer‐review/10.1002/1878‐0261.13530.

## Supporting information


**Fig. S1.** Standards for reporting of diagnostic accuracy studies (STARD) diagram.
**Fig. S2.** UroAmp pre‐IVT high‐risk patients stratified by GDB.
**Table S1.** uCGP predicted recurrence risk.
**Table S2.** AUA/SUO risk categories and outcomes.

## Data Availability

Genome sequencing data used in this study have been deposited in the Sequence Read Archive (SRA) at the National Center for Biotechnology Information (NCBI) and are available through BioProject ID PRJNA973097.
