# Supplementary material for: Urinary comprehensive genomic profiling predicts urothelial carcinoma recurrence and identifies responders to intravesical therapy
Source: Mol Oncol. 2023 Oct 5;18(2):291–304. doi: 10.1002/1878-0261.13530 (PMC10850796; doi:10.1002/1878-0261.13530)
Supplement: Supplementary file 1 — Fig. S1. Standards for reporting of diagnostic accuracy studies (STARD) diagram. Fig. S2. UroAmp pre‐IVT high‐risk patients stratified by GDB. Table S1. uCGP predicted recurrence risk. Table S2. AUA/SUO risk categories and outcomes. [file MOL2-18-291-s001.pdf]

Supporting Information

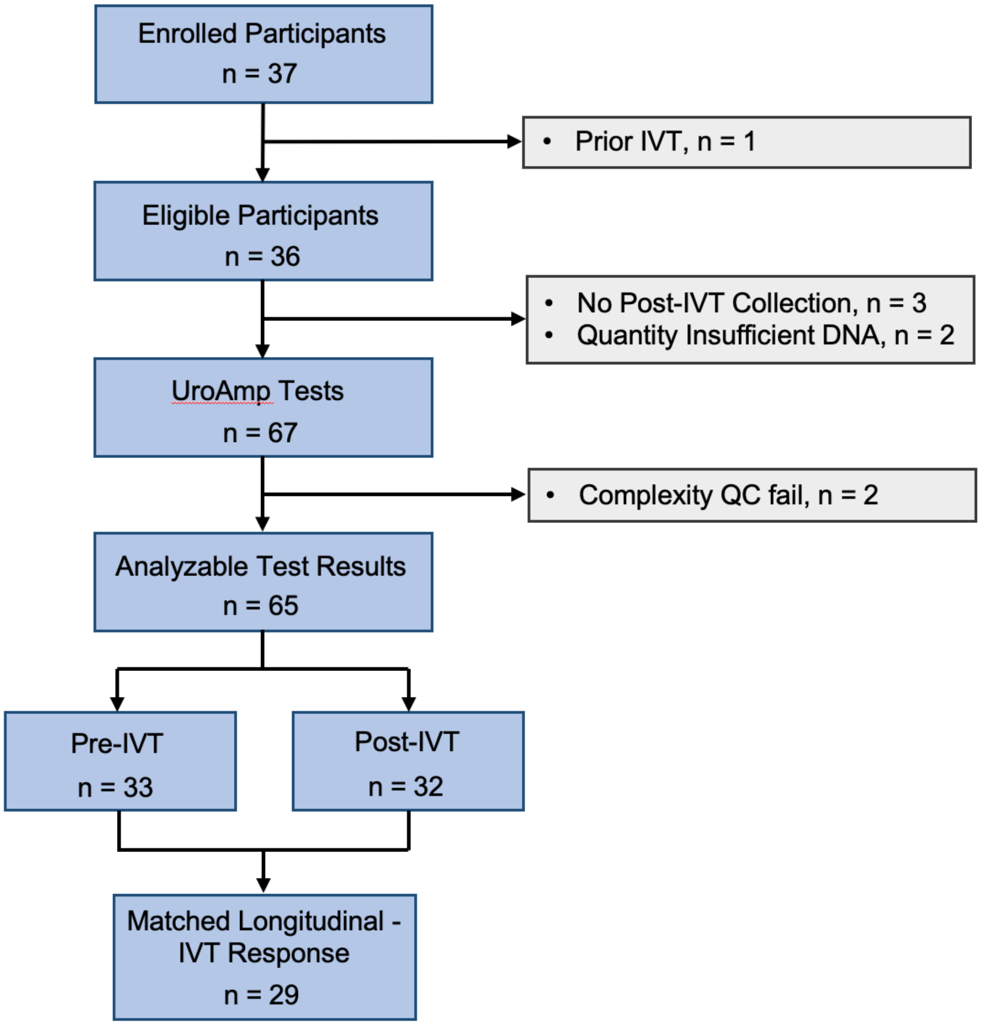

Supplemental Figure 1. Standards for reporting of diagnostic accuracy studies (STARD) diagram. The STARD diagram details specimen usage in this study.

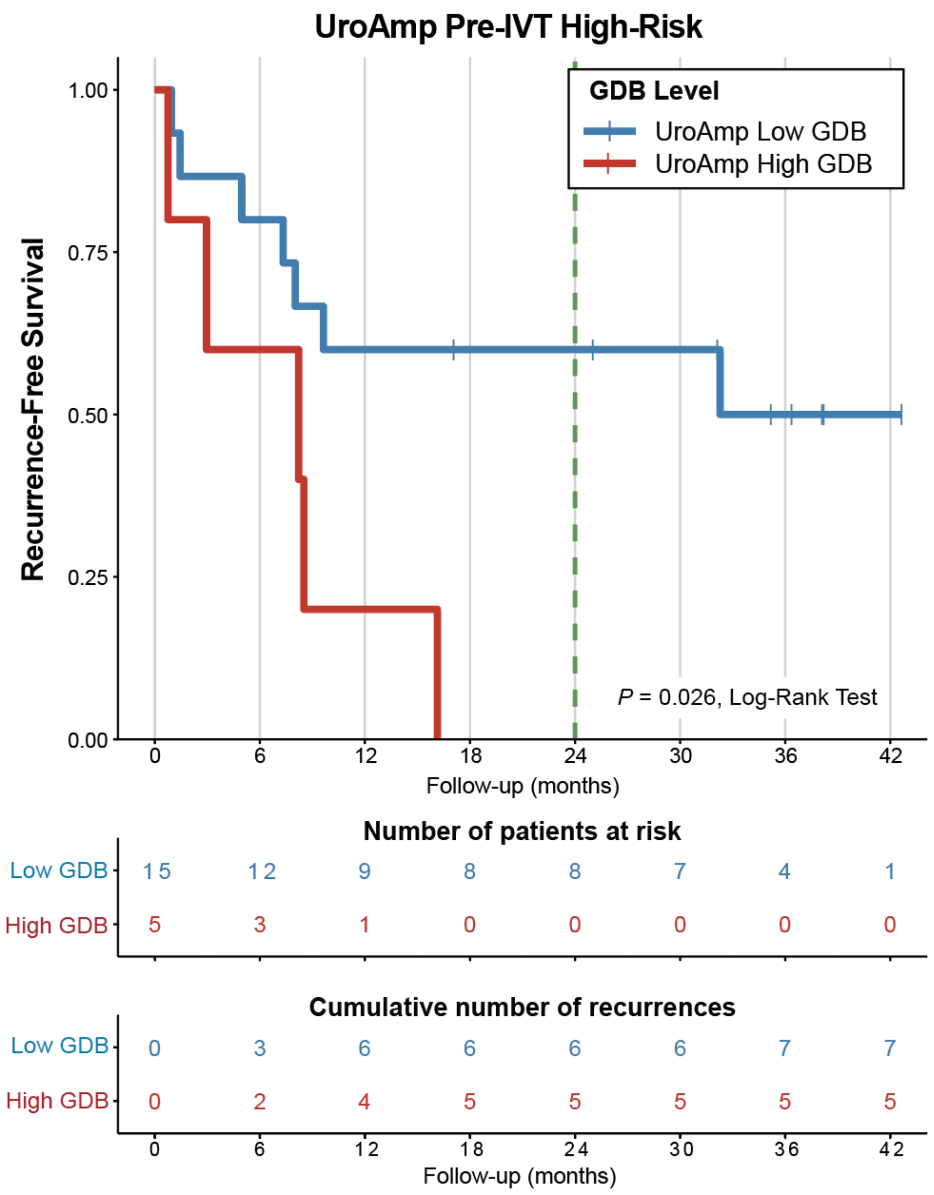

Supplemental Figure 2. UroAmp pre-IVT high-risk patients stratified by GDB. The UroAmp pre-IVT high recurrence risk group was stratified by UroAmp GDB score and recurrence-free survival was calculated. The top quartile of GDB was categorized as High GDB.

Supplemental Table 1. uCGP predicted recurrence risk. Baseline and longitudinal NMIBC cohorts were analyzed for recurrence risk. Univariable Cox proportional-hazard regression analyses of UroAmp recurrence risk and IVT response groups as well as clinical risk factors.

Feature	Predictor	*n*	HR	Confidence Interval	*P*-value
Baseline	UroAmp Low	13	1		
	UroAmp High	20	9.3	[1.21, 71.53]	0.032
IVT Response	MRD Negative	9	1		
	IVT Refractory	12	10.5	[1.29, 84.88]	0.028
	IVT Responder	8	6.5	[0.73, 58.58]	0.093
	IVT Responder + IVT Refractory	20	8.6	[1.12, 66.76]	0.039
Smoking	Never Smoker	11	1		
	Ever Smoker	22	1.6	[0.48, 5.10]	0.464
Age	<70	13	1		
	≥70	20	2.2	[0.60, 7.96]	0.235
Grade	LG	1	1		
	HG	32	0.58	[0.07, 4.51]	0.602
Stage	T1	17	1		
	Ta	13	0.48	[0.12, 1.88]	0.296
	CIS	3	6.4	[1.51, 27.30]	0.012
Other	No CIS	21	1		
	Concomitant CIS	12	1.3	[0.44, 4.12]	0.607

Supplemental Table 2. AUA/SUO risk categories and outcomes. Clinical risk assignment according to AUA/SUO guidelines [1]was broken down by grade, UroAmp risk, and overall recurrence rate.

AUA Risk	Grade	UroAmp Risk	Recurrence Rate
Intermediate (50% recurrence rate)	LG (n = 1)	High (n = 1)	100%
		Low (n = 0)	n/a
	HG (n = 1)	High (n = 0)	n/a
		Low (n = 1)	0%
High (39% recurrence rate)	HG (n = 31)	High (n = 19)	58%
		Low (n = 12)	8%
